# Prediction of Molecular Mechanisms for LianXia NingXin Formula: A Network Pharmacology Study

**DOI:** 10.3389/fphys.2018.00489

**Published:** 2018-05-08

**Authors:** Yang Yang, Kuo Yang, Teng Hao, Guodong Zhu, Ruby Ling, Xuezhong Zhou, Ping Li

**Affiliations:** ^1^The Third Affiliated Hospital, Beijing University of Chinese Medicine, Beijing, China; ^2^Beijing Key Lab of Traffic Data Analysis and Mining, School of Computer and Information Technology, Beijing Jiaotong University, Beijing, China; ^3^Department of Psychiatry, Beijing ChaoYang Hospital of Traditional Chinese Medicine, Beijing, China; ^4^Department of Cardiovascular, Beijing Chaoyang Integrative Medicine Emergency Medical Center, Beijing, China

**Keywords:** NPPA, CRH, network pharmacology, coronary heart disease, LianXia NingXin formula, pharmacological target

## Abstract

**Objectives:** Network pharmacological methods were used to investigate the underlying molecular mechanisms of LianXia NingXin (LXNX) formula, a Chinese prescription, to treat coronary heart disease (CHD) and disease phenotypes (CHD related diseases and symptoms).

**Methods:** The different seed gene lists associated with the herbs of LXNX formula, the CHD co-morbid diseases and symptoms which were relieved by the LXNX formula (co-morbid diseases and symptoms) were curated manually from biomedical databases and published biomedical literatures. Module enrichment analysis was used to identify CHD-related disease modules in the protein–protein interaction (PPI) network which were also associated to the targets of LXNX formula (LXNX formula’s CHD modules). The molecular characteristics of LXNX formula’s CHD modules were investigated via functional enrichment analysis in terms of gene ontology and pathways. We performed shortest path analysis to explore the interactions between the drug targets of LXNX formula and CHD related disease phenotypes (e.g., co-morbid diseases and symptoms).

**Results:** We identified two significant CHD related disease modules (i.e., M146 and M203), which were targeted by the herbs of LXNX formula. Pathway and GO term functional analysis results indicated that G-protein coupled receptor signaling pathways (GPCR) of M146 and cellular protein metabolic process of M203 are important functional pathways for the respective module. This is further confirmed by the shortest path analysis between the drug targets of LXNX formula and the aforementioned disease modules. In addition, corticotropin releasing hormone (CRH) and natriuretic peptide precursor A (NPPA) are the only two LXNX formula target proteins with the low shortest path length (on average shorter than 3) to their respective CHD module and co-morbid disease and symptom gene groups.

**Conclusion:** G-protein coupled receptor signaling pathway and cellular protein metabolic process are the key LXNX formula’s pathways to treat CHD disease phenotypes, in which CRH and NPPA are the two key drug targets of LXNX formula. Further evidences from Chinese herb pharmacological databases indicate that *Pinellia ternata* (Banxia) has relatively strong adjustive functions on the two key targets.

## Introduction

Coronary heart disease (CHD) is a cardiac disease caused by coronary artery arteriosclerosis leading to cardiac vascular stenosis or obstruction, and/or by functional change (or vasospasm) of coronary artery which leads to myocardial ischemia or necrosis (Ni et al., 2016). According to the data published on the World Health Organization website (World Health Organization, 2017), 7.4 million people die from CHD each year. One in seven people die of CHD in United State (Benjamin et al., 2018). CHD has become a serious threat to human life and health and has been a focus of medical research all over the world for a long time. More and more researchers have realized that the development of CHD is related not only to the circulatory system, but also others such as the immune (Cao et al., 2018), nervous (Alcántara and Davidson, 2014), and endocrine systems (Baxter et al., 2003). Therefore, many researchers are now investigating new treatments for CHD by looking for medical targets in systems other than the circulatory system. Around 2000 years ago, traditional Chinese medicine (TCM) had already started and documented research on CHD treatment, as noted in texts such as Synopsis of the Golden Chamber (*Jin Gui Yao Lue*) (Xu et al., 2017). Even today, TCM continues to play an irreplaceable role in CHD treatment. TCM treats CHD in accordance with its holistic principle, and thus, TCM may have already been treating CHD through systems aside from the circulatory system (Zhang et al., 2017).

LianXia NingXin formula (LXNX formula) was created by Professor Ping Li, a Chinese medicine expert in our research team, on the basis of the TCM theory of the heart controlling the blood vessels, nerves and spirit (Yang et al., 2016). It also embodies principles such as TCM master Zhizheng Lu view of “treating CHD via the spleen and stomach” (Song and Lu, 2011), and academician Yongyan Wang’s summation of CHD causes as “poison damaging the veins” (Zhang et al., 2013). LXNX formula targets CHD phlegm-fire syndrome in TCM through the ways to reduce the heat and phlegm in the heart. The results of our previous clinical study (Ma, 2015) showed that after 4 weeks of treatments with LXNX formula, physical activity levels, angina occurrence frequency and degree of pain for CHD patients noticeably improved, while co-morbid symptoms (e.g., palpitation, dizziness, chest pain, chest tightness, insomnia) were relieved significantly. These co-morbid symptoms seem to be related to autonomic nervous system (Cheng et al., 2017). Pre-clinical researches (Li et al., 2015a,b) indicated that LXNX formula decreased tyrosine hydroxylase (TH) mRNA, acetylcholinesterase (AchE) mRNA, nerve growth factor (NGF) mRNA, and tyrosine kinase receptor A (TrKA) mRNA in previous test subjects, mice with myocardial infarction. The results showed that LXNX formula had positive effects on heart sympathetic nerve remodeling and the autonomic nervous system by influencing inflammatory factors, such as NGF (Montoya et al., 2017), and endocrine proteins, such as AchE (Rotundo, 2017). These studies showed that LXNX formula could adjust the nervous, inflammatory, and endocrine systems; LXNX formula’s targets and pathways to treat CHD may also relate to systems other than the circulatory system. Therefore, further research into the exact pharmacological mechanism of action for LXNX formula to treat CHD, co-morbid diseases, and symptoms is helpful for discovering new drugs to improve CHD treatment.

LianXia NingXin formula is composed of nine Chinese herbs including *Coptis chinensis* (*Huang Lian*), Pinellia Tuber (*Ban Xia*), Orange peels (*Chen Pi*), Porio coco (*Fu Ling*), etc. These nine Chinese herbs have more than one hundred drug ingredients which can affect more than one thousand human biological proteins. It will be extremely difficult for us to identify LXNX formula’s drug targets one by one from thousands of proteins through traditional method. In recent years, it is well recognized that human body is a complex system. A drug that only regulates one protein target may not be able to affect the whole human protein network (Lin, 2012). For such reason, the network medicine (Barabási et al., 2011; Zhou et al., 2018) approach for pharmacology was rapidly developed recently. Hopkins presented and elaborated the concept of network pharmacology for the first time (Hopkins, 2008), in accordance with the network pharmacological theory, all the human biological proteins are working as a protein network in which the proteins are the nodes and the interaction relationships between the proteins are the edges. This protein network has many characteristics as a complex network. We can find the interactions between the drug and specific node or module gene group and gain more understanding about the interaction between drug and human body through these characteristics. At present, the analysis of network pharmacology is used in western medicine widely to develop new drugs, to research on the multi-target drug treatment and drug actions, etc. (Tang et al., 2012; Peng et al., 2015). The systemic and synergetic approach of network pharmacology coincides well with the principles of traditional Chinese medicine (e.g., whole body treatment and the treatment mechanism characters of Chinese herbs formulas which had multi-ingredients and influenced multi-targets and multi-pathways) (Liu and Sun, 2012; Zhang Y.Q. and Li X., 2015). Using network pharmacology to analyze herbal prescriptions could result in rapid progress for TCM pharmacology (Li S. et al., 2014; Li, 2016), accelerating translation from an experience-based system into an evidence-based one. Through the preliminary works in this research (Xu et al., 2018), eight disease modules associated to CHD pathogenicity mechanism (CHD modules) have been identified. The identification methods were as follows: Firstly, dividing human protein–protein interaction network (PPI) into 314 topological modules (named from M1 to M314) by community detection algorithm (Dittrich et al., 2008; Wang et al., 2017), which had specific biological function and disease pathogenicity mechanism (Szklarczyk et al., 2011; Menche et al., 2015); secondly, finding the CHD seed gene (Wang et al., 2017; Xu et al., 2018) group composed of the CHD genes derived from public biomedical databases and literatures; thirdly, using the module enrichment analysis between topological modules and the CHD seed gene group to identify eight CHD modules. After that, on the basis of these CHD modules, this study planned to find CHD modules, drug targets, molecule pathways and ingredients related to LXNX formula action mechanism to treat CHD and its co-morbid diseases and symptoms by network pharmacological analysis methods such as disease module analysis, the shortest path analysis, pathway enrichment analysis and gene ontology enrichment analysis (GO term analysis).

## Materials and Methods

### Identifying the Terms Related to CHD Co-morbid Diseases and Symptoms

Coronary heart disease co-morbid diseases and symptoms related to LXNX formula were identified on the basis of the previous pre-clinical and clinical studies on using LXNX formula to treat CHD patients. To obtain the phenotype terms related to LXNX formula, we referred to the NLM’s Medical Subject Heading (MeSH) database^1^ to identify and confirm official terminology of co-morbid diseases and symptoms treatable by LXNX formula.

### Gene Dataset Collection of Co-morbid Diseases and Symptoms

We used the disease terms to filter the associated genes for LXNX formula treated phenotypes from several well-established phenotype-genotype association databases. In particular, genes associated with the co-morbid diseases were identified via the OMIM^2^ and Malacards^3^ databases. Gene information for co-morbid symptoms was gathered from the Malacards, HPO^4^ and DiseaseConnect^5^ databases.

### Construction of Drug Protein Dataset

The drug ingredients and protein targets information associated to nine herbs which constitute the LXNX formula were curated from HIT^6^, TCMID^7^ databases, and the biomedical literatures in PubMed^8^ and CNKI^9^ databases. The data was then organized into an herbs-ingredients-targets relationship dataset for LXNX formula.

### Identifying CHD Modules Related to LXNX Formula

Module enrichment analysis was used between the eight CHD modules determined from the previous preliminary experiment and the following seed gene groups: each individual herb, each co-morbid disease, each co-morbid symptom, set of all herbs, set of all co-morbid diseases, and set of all co-morbid symptoms. Fisher’s exact test was used to calculate the enrichment degree between each seed gene group and the CHD modules. And then, the significant CHD modules related to LXNX formula were identified on the basis of the positive results (*P* < 0.05) of these enrichment results.

### Enrichment Analysis and Shortest Path Analysis of LXNX Formula

Firstly, the functional pathways of CHD module related to LXNX formula were analyzed using pathway and gene ontology (GO) enrichment analysis based on the David platform (Huang et al., 2009). On the platform, the enrichment degree (*p*-value) between CHD modules related to LXNX formula was calculated by Fisher’s exact test. Gene ontology term analysis was based on Gene Ontology database, and was carried out by the plugin BiNGO 2.44 in the software Cytoscape 2.8.2. Pathway enrichment analysis was based on Reactome database and was carried out by online software KOBAS2.072. *P*-value was calculated in these two enrichment analyses, and *P* < 0.05 means the enrichment degree had statistically significance and the pathway results would be important functional mechanisms of disease modules. Secondly, the drug protein targets of LXNX formula in CHD modules related to LXNX formula were identified through shortest path analysis. The first step of the shortest path analysis is to calculate the shortest path between any node pair in the PPI network; subsequent calculations are then done to find the average shortest path length of a fixed source node to given node set. The shorter the average length is, the more important this source node is in connecting the entire node set. The Dijkstra algorithm (Dijkstra, 1959; Misa and Frana, 2010), which is the most classic algorithm to calculate shortest path of networks, was used to calculate the shortest path length of PPI network. In this study, we conducted the shortest path analysis between protein targets of LXNX formula and the genes of LXNX formula’s CHD gene modules, and the genes of all co-morbid diseases or symptoms. The average shortest path length for strongly connecting nodes was defined as less than 3. The nodes which were found by the shortest path analysis were the key drug targets of LXNX formula in CHD modules of LXNX formula or all co-morbid diseases or all co-morbid symptoms. Finally, we identified the CHD modules, key drug targets and molecular pathways related to LXNX formula for CHD treatment.

### Candidate Targets Prediction of LXNX Formula

**FIGURE 1:**
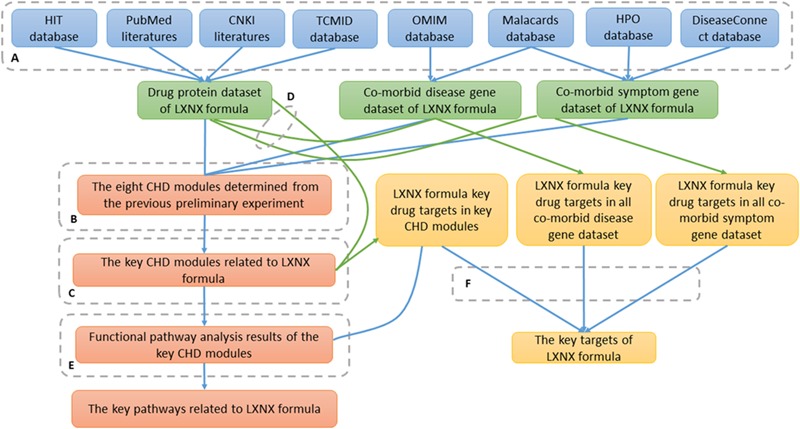
A flowchart of the pathway analysis and targets identification of LXNX formula. To obtain reliable results, the following steps were taken: **(A)** The genetic information of LXNX formula herb ingredients, co-morbid diseases and symptoms was acquired from open biological gene databases. **(B)** For determining the key CHD modules related to LXNX formula, module enrichment analysis was used between each CHD module determined from the previous preliminary experiment and the following seed gene groups: each individual herb, each co-morbid disease, each co-morbid symptom, set of all herbs, set of all co-morbid diseases, and set of all co-morbid symptoms. **(C)** The function pathways of the key CHD modules of LXNX formula were determined using pathway and GO term enrichment analysis. **(D)** Using shortest path analysis, the key drug targets of LXNX formula in the key modules, respectively, all co-morbid diseases and symptoms were determined. Green lines showed the process of shortest path analysis. **(E)** LXNX formula key pathways to treat CHD were determined based on the results of module functional pathway analysis and LXNX formula key targets in the modules. **(F)** LXNX formula key drug targets were determined based on the results of shortest path analysis.

Similarly, we identified the drug targets of LXNX formula in regards to gene sets of co-morbid diseases and symptoms using shortest path analysis, respectively. Based on these drug targets and the targets for LXNX formula’ s CHD modules, key candidate drugs targets of LXNX formula to treat both CHD and its co-morbid diseases and symptoms were identified. **Figure 1** showed the flowchart of the pathway analysis and target identification of LXNX formula to treat CHD.

## Results

### CHD Related Disease Phenotype MeSH Terms

The results of previous pre-clinical studies (Li J. et al., 2014; Li et al., 2015a,b; Zhang H.N. and Li P., 2015) on LXNX formula indicated that LXNX formula had good effects on heart sympathetic nerve remodeling, arrhythmia and autonomic nerve system. By comparing the symptoms before and after the patients were treated with LXNX formula, clinical research (Ma, 2015) showed that LXNX formula can relieve CHD co-morbid symptoms significantly (i.e., chest pain, shortness of breath, palpitation, thirst, bitter taste in mouth, dizziness, chest tightness, and dream-disturbed sleep). It can also significantly relieve anxiety and insomnia. Based on the searching results from MeSH database, eight MeSH headings for five co-morbid diseases and ten Mesh headings for nine co-morbid symptoms were found in total (see **Table 1**).

**Table 1:** The MeSH terms and number of genes related to LXNX formula co-morbid diseases and symptoms.

Disease or symptom name	Medical subject heading	Number of related genes
Anxiety (D)	Anxiety	248
Arrhythmia (D)	Arrhythmia	167
Autonomic dysfunction (D)	Autonomic dysfunction	7
Cardiac sympathetic remodeling (D)	Cardiac sympathetic innervation	1
	Cardiac sympathetic innervation remodeling	1
	Cardiac sympathetic innervation remodeling heart	3
Insomnia (D)	Insomnia	22
	Sleep disorder	19
Chest pain (S)	Chest pain	1624
Dizziness (S)	Dizziness	1761
Insomnia (S)	Insomnia	248
Palpitations (S)	Palpitations	103
Shortness of breath (S)	Shortness of breath	176
Thirst (S)	Thirst	225
Chest tightness (S)	Chest tightness	0
	Chest discomfortable	0
Bitter taste in mouth (S)	Bitter taste in mouth	0
Dream-disturbed sleep (S)	Dream-disturbed sleep	0

*In the disease or symptom column, (D) indicates co-morbid disease, while (S) means co-morbid symptoms. In the column of number of related genes, the numbers mean the count number of genes related to medical subject heading on left.*

### CHD-Co-morbid Diseases and Symptoms Related Genes

In accordance with the searching results from gene databases, there were 3230 genes related to LXNX formula co-morbid diseases and symptoms. These genes had 4605 interactions with co-morbid diseases and symptoms. For the symptoms of chest tightness, bitter taste in mouth, and dream-disturbed sleep, no related genes were found (see **Table 1** and Supplementary Tables S1, S2).

### The Herb Targets of LXNX Formula

LianXia NingXin formula ingredients and their corresponding targets information had been curated from various databases and documents with the metadata like data sources, curated time and keywords are kept as well. Only one record had been kept if there were any records with the same information about Chinese herb ingredients and targets. Finally, 134 chemical ingredients were identified from the nine herbs included in LXNX formula. These chemical ingredients were noted to have influence on 1860 human biological proteins (with totally 3424 protein interactions) (see Supplementary Table S3).

### Key CHD Modules Related to LXNX Formula

Module enrichment analysis between the preliminary eight CHD modules and various seed gene groups for co-morbid diseases, co-morbid symptoms, and Chinese herbs showed that M146 and M203 had statistically significant results (*p* < 0.05). Specifically, M146 had 12 positive results, while M203 had 8 positive results (see **Table 2**).

**Table 2:** The positive result summary of module enrichment analysis.

Module ID	Seed gene group	Number of genes	*p*-value	Proportion
146	Anxiety (D)	13	2.90E-06	4.770082
146	Chest pain (S)	25	0.0221	1.38308
146	Dizziness (S))	28	0.012776	1.429755
146	Insomnia (S)	8	0.00495	2.877702
146	Palpitations (S)	12	1.27E-09	10.55632
146	Shortness of breath (S)	5	0.033574	2.538544
146	Thirst (S)	23	5.05E-16	9.163804
146	Pinellia Tuber(Ban Xia)	25	1.40E-05	2.487102983
146	Poria coco(Fu Ling)	8	0.041464852	1.821802935
146	Diseases	14	0.000434	3.083373
146	Herbs	34	0.002613	1.809648
146	Symptoms	57	9.68E-06	2.129997
**M146**	**Positive results**			**12**
203	Arrhythmia (D)	3	0.005047	8.311160088
203	Autonomic dysfunction (D)	1	0.015491	63.71889401
203	Chest pain (S)	8	0.013648	2.270036
203	Dizziness (S)	9	0.00725	2.357119
203	Palpitations (S)	2	0.019188	9.023954
203	Thirst (S)	3	0.011237	6.130623
203	Diseases	5	0.002451	6.064949
203	Symptoms	13	0.006585	2.732289
**M203**	**Positive results**			**8**
194	Chest pain (S)	9	0.000671	3.298646
194	Dizziness (S)	9	0.001176	3.044612
194	Insomnia (S)	3	0.007411	7.149291
194	Symptoms	12	0.001491	3.783921
**M194**	**Positive results**			**4**
195	Chest pain (S)	4	0.049971	2.513254
195	Symptoms	7	0.014961	3.779208
**M195**	**Positive results**			**2**
204	Cardiac sympathetic remodeling (D)	1	0.049286	19.47464789
204	Dizziness (S)	24	0.025127	1.37222
**M204**	**Positive results**			**2**
95	Chest pain (S)	3	0.020438	4.398194
**M95**	**Positive results**			**1**

*The positive result summary of module enrichment analysis between 8 CHD modules and the seed gene groups for Chinese herbs, co-morbid diseases, and co-morbid symptoms, calculated both collectively and separately. In modular ID column, the numbers refer to CHD module number. In the seed gene group column, (S) denotes single co-morbid symptom while (D) denotes single co-morbid disease, “diseases” denotes all co-morbid diseases, “herbs” denotes all LXNX formula herbs, and “symptoms” denotes all co-morbid symptoms. In the positive results rows, the number shown means positive result count number related to that specific CHD module shown on the left.*

### Pathway and GO Term Enrichment Analysis

There were 46 gene pathways related to M146 and 2 gene pathways related to M203 according to pathway enrichment analysis (*P* < 0.05) (see Supplementary Tables S7, S9). There were 119 gene pathways related to M146 and 29 gene pathways related to M203 according to GO term enrichment analysis (see Supplementary Tables S6, S8). Based on the results of shortest path analysis between each LXNX formula’s CHD modules and LXNX formula’s protein targets, there were 30 key drug targets of LXNX formula in M146 (see Supplementary Table S10), and 3 key drug targets of LXNX formula in M203 (see Supplementary Table S11). The G-protein coupled receptor (GPCRs) signaling pathway that M146 enriches contained more key drug targets of LXNX formula in the M146 than other enriched pathways. Cellular protein metabolic process, which was one of the M203 GO enrichment, contained all key drug targets of LXNX formula in the M203. Other functional pathway enrichment results in M203 did not contain as many key drug targets. **Table 3** shows the functional pathway results of GPCRs signaling pathway related to M146, and specific cellular protein metabolic process related to M203 based on the pathway enrichment analysis and GO term enrichment analysis.

**Table 3:** The functional pathway analysis of CHD modules related to LXNX formula.

Modular ID	Category	Term	Number of pathway gene	Number of key protein
146	KEGG_PATHWAY	hsa04080:Neuroactive ligand-receptor interaction	42	17
146	KEGG_PATHWAY	hsa04024:cAMP signaling pathway	29	14
146	GOTERM_BP_DIRECT	GO:0007189∼adenylate cyclase-activating G-protein coupled receptor signaling pathway	34	20
146	GOTERM_BP_DIRECT	GO:0030819∼positive regulation of cAMP biosynthetic process	30	10
146	GOTERM_BP_DIRECT	GO:0007190∼activation of adenylate cyclase activity	27	17
146	GOTERM_BP_DIRECT	GO:0007186∼G-protein coupled receptor signaling pathway	56	15
146	GOTERM_BP_DIRECT	GO:0007188∼adenylate cyclase-modulating G-protein coupled receptor signaling pathway	19	10
146	GOTERM_BP_DIRECT	GO:0006171∼cAMP biosynthetic process	14	10
146	GOTERM_BP_DIRECT	GO:0019933∼cAMP-mediated signaling	14	10
146	GOTERM_BP_DIRECT	GO:0007193∼adenylate cyclase-inhibiting G-protein coupled receptor signaling pathway	13	9
203	GOTERM_BP_DIRECT	GO:0044267∼cellular protein metabolic process	16	3

*In the module ID column, the numbers denote numbers of CHD modules related to LXNX formula. In the category column, KEGG-PATHWAY refers to the pathway enrichment analysis, and GOTERM_BP_DIRECT refers to the GO term enrichment analysis. In the term column, the text indicates results of function enrichment analysis corresponding to the type of analysis shown on the left. In the number of pathways gene column, the numbers refer to the gene count numbers corresponding to the pathway on the left. In the number of key protein column, the numbers refer to the protein count numbers of key drug targets in the corresponding pathway on left.*

### Molecular Interactions Between Herb Targets and Modules

Thirty proteins in LXNX formula were closely related to M146 genes (see Supplementary Table S10), while 3 proteins were related to M203 genes (see Supplementary Table S11), 144 proteins were related to all co-morbid symptom genes (see Supplementary Table S5), 29 proteins were related to whole co-morbid disease genes (see Supplementary Table S4). There was no single protein in LXNX formula associated with both M146 and M203. Among LXNX formula proteins, corticotropin releasing hormone (CRH) was the key drug target simultaneously in M146 and all co-morbid diseases and symptoms. In addition, natriuretic peptide precursor A (NPPA) was the key drug target simultaneously in M203 and all co-morbid diseases and symptoms (see **Table 4**). Besides CRH and NPPA, there were no other proteins which served as key drug targets simultaneously in LXNX formula’s CHD modules and all co-morbid diseases and symptoms.

**Table 4:** The shortest path analysis results about CRH and NPPA.

Herb target	Gene network	Min_sp	Max_sp	Avg_sp
CRH	Diseases	0	7	2.943452
	Symptoms	0	9	2.998833
	M146	0	4	1.686667
NPPA	Diseases	0	6	2.705357
	Symptoms	0	9	2.740698
	M203	0	3	1.548387

*In herb target column, the symbols indicate the key targets of LXNX formula in M146 and M203, respectively. In gene network column, diseases refer to the gene network consisted of genes related to all co-morbid diseases, symptoms refer to the gene network consisted of genes related to all co-morbid symptoms, M146 and M203 refer to the gene network included the genes related to M146 and M203, respectively. In min_sp column, the numbers refer to the minimum shortest path length. In max_sp column, the numbers refer to the maximum shortest path length. In avg_sp column, the numbers refer to the average shortest path length.*

**FIGURE 2:**
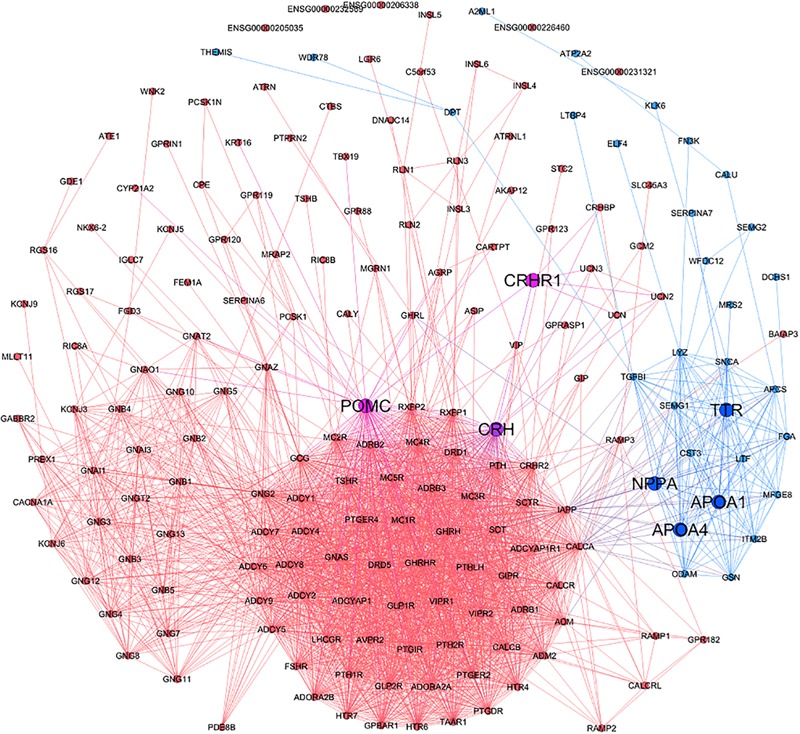
The protein interactions of M146 and M203. The red and blue nodes represent the proteins of M146 and M203, respectively. The red and blue lines represent the protein interactions of M146 and M203, respectively.

**Figure 2** showed the gene relationships between M146 and M203 in the human protein–protein interaction network, and it also reflected the key targets (those big nodes in the figure) and pathways (those bold lines between key targets) of LXNX formula to treat CHD based on the analysis results of the enriched functional pathway and the shortest path.

## Discussion

Presently, the incompleteness of knowledge regarding human protein interaction and genes related to diseases resulted in the hindering of research into complex systemic diseases such as CHD. Recently, it has been recognized that proteins associated with diseases did not exist randomly in a scattered network, but rather were connected into functional subgroups called disease modules. Thus, disease module analysis can now be used to identify previously unknown genes and mechanisms associated with complex diseases (Vinayagam et al., 2016; Mukund and Subramaniam, 2017). For this study, protein–protein interaction (PPI) networks, the most intensely studied biology network (Zhou et al., 2014), were used to determine disease modules. By consulting open bioinformatics databases and using module enrichment analysis, disease modules related to different diseases, symptoms, and herbs were identified based on the gene groups of these different networks. Results with significant enrichment (*p* < 0.05) indicated that the biological mechanism of the disease module was related to the functional pathway of the disease, symptom, or herb seed gene groups. Disease module analysis results showed that the functional pathways in M146 and M203 were strongly associated with these aforementioned seed gene groups, both individually and collectively. Other CHD modules did not have such lots of positive results. Therefore, these results indicate that the treatment effect of LXNX formula for CHD and its co-morbid diseases and symptoms is mainly accomplished by modulating M146 and M203 functional pathways.

Based on the results of pathway and GO term enrichment analysis and of the shortest path analysis, the signaling pathways of G-protein coupled receptors (GPCRs) related to adrenaline, adenylate cyclase, dopamine, and calcium ion are significant in M146, and the drug targets of LXNX formula in M146 are all related to these pathways; the pathways of specific cellular protein metabolic process related to the apolipoprotein (APO) and natriuretic peptide are important in M203, and the drug targets in M203 are all related to these pathways. GPCRs signaling transduction pathway constitute a large protein family of cellular surface receptors which are responsible for various ligand responses, signal transduction from extracellular space to intracellular space, and activating cellular responses. GPCRs are largely expressed in human body, and play a key role in regulation. For example, β-adrenergic receptor (β-AR) is a typical ligand activated GPCR. Afterβ-AR is activated, adenylate cyclase is then activated, and the concentration of cyclic adenosine monophosphate (cAMP) is subsequently increased, inducing a series of biological effects. β-AR is widely distributed in the heart and thus plays an important role in regulating cardiac functions (Mahoney and Sunahara, 2016; Gurevich and Gurevich, 2017). The pathways of cellular protein metabolic process may control cell apoptosis or proliferation by adjusting the synthesis or decomposition of the proteins in cells, thus regulating the repair and necrosis of nerves and vascular smooth muscle. For example, apolipoprotein A1 (APOA1), apolipoprotein A4 (APOA4) may impact low-density lipoprotein (LDL) particle density (Gomez et al., 2010). LDL is a protein in the cell. LDL particle density is an important factor in atherosclerosis. In accordance with the module function analysis, the shortest path analysis results between LXNX formula protein targets and LXNX formula’s CHD module gene networks, respectively, and the mechanism characters of the pathway, the two pathogenic pathways, GPCRs signaling pathway and cellular protein metabolic process, both have three same characters. Firstly, they are both the important pathogenic pathways of LXNX formula’s CHD module, respectively. Secondly, they both are composed of a lot of LXNX formula’s important drug targets in, respectively, CHD modules of LXNX formula. Thirdly, they can influence CHD and CHD co-morbid diseases which can be treated by LXNX formula. Hence, GPCRs signaling pathway and specific cellular protein metabolic process both are important pathways of LXNX formula to treat CHD and its co-morbid diseases and symptoms based on the three characters of these two pathway mentioned above.

The proteins CRH and NPPA are critical drug targets for LXNX formula’s CHD module and all co-morbid diseases and symptoms; no other protein has shown such simultaneous results. CRH is synthesized in the hypothalamus and acts upon the hypothalamus-pituitary-adrenal gland axis (HPA axis). HPA axis is a complex system which includes direct effects and negative feedback mechanisms. When stressed (a physical reaction that occurs in imbalanced circumstances or when facing threats to internal stability), the hypothalamus secretes CRH (Zhou et al., 2018), which proceeds to combine with CRH receptor 1 (CRHR1) to promote the release of adreno-corticotropic-hormone (ACTH). ACTH then acts upon the adrenal gland to stimulate the release of glucocorticoid (GC), which has a negative feedback effect on the hypothalamus and pituitary glands (Asalgoo et al., 2017; Paragliola et al., 2017). Thus, HPA axis is an important part of the neuroendocrine system and is essential to controlling the effects of stress. The stimulation of chronic stress will lead to constant HPA axis excitation. It will cause disorders of the endocrine system, imbalanced physiological functions, and mental disorders (Contoreggi, 2015). The increase of GC synthesis is a key factor in HPA axis mechanism; the constant increase of GC promotes immune system activity and inflammatory response, injures vascular endothelial cells, and accelerates arteriolosclerosis (AS) (Deuchar et al., 2011; Cho et al., 2015). CRH causes the HPA axis to release GC by controlling ACTH release; therefore, CRH can affect CHD. There are three types of CRH receptors, CRH-R1, CRH-R2, and CRH-R3, which all belong to G-protein coupled receptors. After CRH is combined with CRH receptors, G protein activates adenylate cyclase, resulting in increased cAMP cell concentration and protein kinases A (PKA) activation. The CRH function that affects CHD is achieved through GPCRs signaling pathway (Inda et al., 2017). ACTH is a proopiomelanocortin (POMC) derivative (Parvin et al., 2017). CRH is the key target of LXNX formula in M146. CRHR1 and POMC are genes not only belonging to M146, but also belonging to GPCRs signaling transduction pathway based on the results of the GO term and pathway functional enrichment analysis. Therefore, LXNX formula’s mechanism of treating CHD and co-morbid diseases and symptoms could be as such: LXNX formula acts upon CRH, and then influences the HPA axis through GPCRs signaling transduction pathway related to CRHR1 and POMC.

Natriuretic peptide precursor A is the precursor to atrial natriuretic peptide (ANP) before synthesis; NPPA polymorphism may induce myocardial necrosis, hypertension, atrial fibrillation, water and sodium disequilibrium, and heart remodeling (Lynch et al., 2009; Francia et al., 2013). After ANP combines with the natriuretic peptide receptor A (NPR-A), cGMP in cell is adjusted, thus influencing myocardial contractions and hindering the proliferation of vascular smooth muscle cells (Song et al., 2015). ANP takes part in the metabolism of the endocardium macrophage inflammatory proteins, such as tumor necrosis factor-α(TNF-α), and interleukin-1 (IL-1). These inflammatory factors can lead to AS plaque occurrence and development, thus affecting the pathological development of AS diseases, including coronary heart disease (Clarke et al., 2010; Kleinbongard et al., 2010). ANP has two pathways to adjust macrophage protein metabolism. First, ANP adjusts the metabolism and synthesis of macrophage inflammatory factors directly through cyclic guanosine monophosphate (cGMP) (Kiemer et al., 2005; Howlett and Moore, 2006). Second, ANP interaction with amyloid factors, such as APOA1, APOA4, and transthyretin (TTR), adjusts macrophage protein metabolism by affecting amyloidosis deposits in coronary artery (Maioli et al., 2000; Torricelli et al., 2004a,b). NPPA, APOA1, APOA4, and TTR are the genes belonging to both M203 and the cell protein metabolic pathway which is one significant result of the M203 GO term analysis. Therefore, LXNX formula mechanism to treat CHD is related to NPPA and the pathway of macrophage protein metabolism containing APOA1, APOA4 and TTR.

In summary, CRH and NPPA have strong relationship with LXNX formula for the reasons that they are both the most important drug targets of LXNX formula in LXNX formula’s CHD modules, respectively, co-morbid diseases and symptoms, they are both relevant to the important pathways, respectively, of LXNX formula, and they both can have influence on CHD and CHD co-morbid diseases which LXNX formula can treat. Therefore, CRH and NPPA are key drug targets of LXNX formula to treat CHD and its co-morbid diseases and symptoms.

Based on the herb-ingredient-target relationship database for LXNX formula, gamma-aminobutyric acid (GABA) found in *Pinellia ternata* (*Ban Xia*) has a direct influence on CRH; adenine in Porio coco (*Fu Ling*), undecanal in dried orange peels (*Chen Pi*), guanosine and L-arginine (L-Arg) in *Pinellia ternata* (*Ban Xia*) have a strong impact on NPPA. Evidently, *Pinellia ternata* (*Ban Xia*), Porio coco (*Fu Ling*), and dried orange peels (*Chen Pi*) are important herbs in LXNX formula. GABA is an important inhibitory neurotransmitter in the brain. By combining with GABA receptors, it inhibits CRH neuron excitability to reduce the release of CRH (Kakizawa et al., 2016). L-Arg is the precursor of EDRF (Endothelium derived relaxing factors)/NO. Shortage of EDRF/NO is essential to the occurrence of cardiovascular diseases including CHD (Charpie et al., 1997). On the other hand, L-Arg decreased ANP gene expression of mice with myocardial hypertrophy caused by isoprenaline (Shen et al., 2004).

Based on the analysis mentioned above about previous documentation, GPCRs signaling transduction pathway related to CRH and cellular protein metabolic process pathway related to NPPA both can influence immune and nervous systems to treat CHD, this character is accordance with the mechanism of LXNX formula to treat CHD. Thus, the forecast results of network medicine concerning LXNX formula’s mechanism of action for treating CHD is reliable. In future research, further experimentation can be done to test the influence of LXNX on CRH and NPPA pathway by *in vitro* and *in vivo* experiments on mice with myocardial ischemia.

## Conclusion

In accordance with this research, the important pharmacological mechanisms of LXNX formula to treat CHD disease phenotypes are that the ingredients such as L-arginine and gamma-aminobutyric acid, etc. in Pinellia Tuber, Poria coco, affect CRH in GPCRs signaling pathway and NPPA in specific cellular protein metabolic process.

## Author Contributions

PL, XZ, YY, and KY conceived and designed the study. YY, TH, and GZ extracted the data. KY analyzed the data. PL, XZ, YY, KY, and RL wrote and revised the manuscript. All authors reviewed and approved the submitted version of the manuscript.

## Conflict of Interest Statement

The authors declare that the research was conducted in the absence of any commercial or financial relationships that could be construed as a potential conflict of interest.

## Footnotes

1 http://www.nlm.nih.gov/mesh/MBrowser.html

2 http://www.omim.org

3 http://www.malacards.org

4 http://human-phenotype-ontology.github.io/

5 http://disease-connect.org/

6 http://lifecenter.sgst.cn/hit

7 http://www.megabionet.org/tcmid/search/

8 www.pubmed.com

9 http://www.cnki.net
